# Treatment of Skeletal Muscle Injury: A Review

**DOI:** 10.5402/2012/689012

**Published:** 2012-04-26

**Authors:** L. Baoge, E. Van Den Steen, S. Rimbaut, N. Philips, E. Witvrouw, K. F. Almqvist, G. Vanderstraeten, L. C. Vanden Bossche

**Affiliations:** ^1^Beijing Tiantan Hospital, Capital Medical University, Beijing Tiantan Xili 6, Beijing 100050, China; ^2^Department of Physical and Rehabilitation Medicine, Ghent University Hospital, 9000 Ghent, Belgium; ^3^Department of Rehabilitation Sciences and Physiotherapy, Ghent University, 9000 Ghent, Belgium; ^4^Department of Orthopaedic Surgery and Traumatology, Ghent University Hospital, 9000 Ghent, Belgium

## Abstract

Skeletal muscle injuries are the most common sports-related injuries and present a challenge in primary care and sports medicine. Most types of muscle injuries would follow three stages: the acute inflammatory and degenerative phase, the repair phase and the remodeling phase. Present conservative treatment includes RICE (rest, ice, compression, elevation), nonsteroidal anti-inflammatory drugs (NSAIDs) and physical therapy. However, if use improper, NSAIDs may suppress an essential inflammatory phase in the healing of injured skeletal muscle. Furthermore, it remains controversial whether or not they have adverse effects on the healing process or on the tensile strength. However, several growth factors might promote the regeneration of injured skeletal muscle, many novel treatments have involved on enhancing complete functional recovery. Exogenous growth factors have been shown to regulate satellite cell proliferation, differentiation and fusion in myotubes in vivo and in vitro, TGF-*β*1 antagonists behave as inhibitors of TGF-*β*1. They prevent collagen deposition and block formation of muscle fibrosis, so that a complete functional recovery can be achieved.

## 1. Introduction

Skeletal muscle injuries are the most common sports-related injuries and present a challenge in primary care and sports medicine. Athletes sustain muscle injuries through a variety of mechanisms, including direct trauma (e.g., lacerations, strains, and contusions) and indirect injuries (related to ischemia and neurological dysfunctions). 

A regeneration process that is similar in most types of muscle injuries, has been observed. However, complete recovery from the injury is compromised due to the development of fibrosis in the second week after the injury. The formed scar tissue always is mechanically inferior and therefore much less able to perform the functions of a normal muscle fiber. It is also more susceptible to reinjury [1, 2]. To minimize the disability and enhance full functional recovery after skeletal muscle injuries, the current conservative treatment includes limiting the bleeding with compression, elevation, and local cooling, nonsteroidal anti-inflammatory drugs (NSAIDs), and physical therapy [3]. 

Recently, it has been suggested that growth factors might promote the regeneration of injured skeletal muscle, and many novel treatments have been developed. 

This review paper focuses on therapeutic approaches including new knowledge of routine NSAIDs, novel biological repair, and physical therapy. A search of the literature on the treatment of skeletal muscle injuries was conducted using PubMed and Medscape. 

## 2. The Pathological Process Following Muscle Injury

The general injury and repair mechanism is similar in most types of muscle injuries. Three stages are distinguished: the destruction and inflammatory phase (1 to 3 days), the repair phase (3 to 4 weeks), and the remodeling phase (3 to 6 months) [4, 5]. The last two phases tend to overlap. 

When a muscle is injured, the myofibers rupture and necrotize. A haematoma is formed. At the same time during this first phase, the inflammatory cells can freely invade the injury site because the blood vessels are torn. The most abundant inflammatory cells are the polymorphonuclear leukocytes. These are replaced by monocytes, a few hours after the injury. These cells eventually transform into macrophages. Macrophages have 2 functions. Firstly, they remove the necrotic myofibers by phagocytosis. Secondly, they produce, together with fibroblasts, chemotactic signals such as growth factors, cytokines, and chemokines. The extracellular matrix (ECM) also contains growth factors that become active when tissue is damaged. Some of these growth factors, such as FGF (fibroblast growth factor), IGF-1 (insulin-like growth factor-1), IGF-2 (insulin-like growth factor-2), TGF-*β* (transforming growth factor-*β*), HGF (hepatocyte growth factor), TNF-*α* (tumor necrosis factor-*α*), and IL-6 (interleukin-6) can activate myogenic precursors, called the satellite cell [3, 6, 7].

The next phase, the repair phase, consists of 2 concomitant processes. The first is the regeneration of the disrupted myofibers. Regeneration can occur because there still is a pool of undifferentiated reserve cells, also called myogenic precursors or satellite cells under the basal lamina of the myofiber. The satellite cells will proliferate and eventually differentiate into myoblasts. Because these new myoblasts fuse with the injured myofibers, the gap formed between the two ends of the injured myofiber is refilled. The second process of the repair phase is the formation of a connective tissue scar by fibrin and fibronectin, derived from blood of the haematoma that was formed immediately after the injury. The scar tissue gives the muscle strength to withstand contractions, and it gives the fibroblasts an anchoring site to invade the granulation tissue. However, in case of excessive proliferation of these fibroblasts, dense scar tissue is formed within the injured muscle. This not only interferes with the repair process but also interrupts the muscle regenerative process and contributes to incomplete functional recovery of the injured muscle during the third phase, the remodeling phase. In this last phase, the newly formed myofibers mature. At the same time, the scar tissue is reorganized and it contracts [3, 6, 7].

Due to an injury, the intramuscular nerve branches can be damaged. Hence, the muscle fibers may be denervated, which might affect the healing process negatively [8].

The whole process is coordinated through different mechanisms like cell-cell and cell-matrix interactions as well as extracellular secreted factors. HGF, IL-1, and IL-6 are secreted factors that can stimulate the activity of satellite cells. FGF and IGF can also activate satellite cells, but in contrast to IGF, FGF can also inhibit their differentiation, while IGF stimulates the differentiation. TGF-*β*1 stimulates collagen deposition, leading to the formation of fibrotic scar tissue [9–13]. 

## 3. Therapeutic Strategies

A variety of conservative treatment strategies exist for acute and chronic skeletal muscle injuries [14, 15]. The primary treatment goals are to minimize further damage, relieve pain and spasm, reduce haemorrhage and edema, and promote healing. Furthermore, the recurrent nature of muscle injuries often requires a functional approach from the acute phase to the final goal of return to sports.

## 4. RICE

The best known treatment immediately after a muscle injury is the “RICE approach”. This acronym stands for rest, ice, compression and elevation. The aim is to minimize the haematoma of the injured muscle and, subsequently, the size of the connective tissue scar. However, the effectiveness of this approach has not been proven in any randomized clinical trial [3]. Ice should be applied intermittently for 15 to 20 minutes with an interval of 30 to 60 minutes. Longer periods of cold application lead to increased circulation and increased bleeding [8].

## 5. Physiotherapy

Early mobilization accelerates capillary ingrowth and promotes the regeneration of muscle fibers. The healed muscle also more rapidly regains its preinjury level of strength. However, early mobilization also has disadvantages. The scar that is formed will be larger, and reruptures will be more common. Therefore, rest is advised during the first 3 to 7 days to allow the scar tissue to gain strength. Subsequently, mobilization within the painfree limits is initiated. Continued inactivity can lead to atrophy of the healthy muscles, excessive deposition of connective tissue within the muscles and a substantially retarded recovery of the strength of the injured skeletal muscle. Exercises should be started gradually. Isometric training should be followed by isotonic training and isotonic training by isokinetic training once the respective exercises can be performed without pain [3].

## 6. NSAIDs

NSAIDs are primarily used for their analgesic, anti-inflammatory, and antipyretic properties [16]. Inflammatory cells play an important role in the healing process of an injured muscle. Therefore, the use of drugs that inhibit these cells, such as NSAIDs, is questioned nowadays. Experimental studies in which NSAIDs were given immediately after the injury, have shown conflicting results. NSAIDs would not have a greater effect on the pain of a muscle injury than paracetamol, but they have more side effects including asthma exacerbations, gastrointestinal and renal side effects, hypertension, and other. However, NSAIDs also have beneficial effects. The inflammatory process can be excessive and cause edema, resulting in anoxia and further cell death. This can be prevented by the administration of low-dose NSAIDs [17].

Rahusen et al. reviewed earlier reports on the use of NSAIDs to clarify recommendations for their use [18]. Basically, NSAIDs should be given no sooner than 48 hours following exercise-induced muscle injuries to provide analgesia and to reduce the early inflammatory response. Earlier use can interfere with the cell chemotaxis that is necessary for the repair and remodeling of regenerating muscle. In the 2 days after the injury, paracetamol can be used for analgesia. Prolonged use of NSAIDs (over 7 days) is not recommended as it would delay muscle regeneration by inactivating the proliferation and differentiation of satellite cells and inhibiting the production of growth factors [18, 19].

 It would also reduce the biomechanical strength of the injured muscle and delay elimination of the haematoma and the necrotic tissue [20]. In contrast with the findings of these authors, Engelberg et al. and Almekinders [21] showed no significant effect on tensile strength recovery following NSAID treatment for muscle strain injury. Engelberg et al. further demonstrated that muscle strength also remained unaltered [22]. 

## 7. Biological Repair

**Figure 1:**
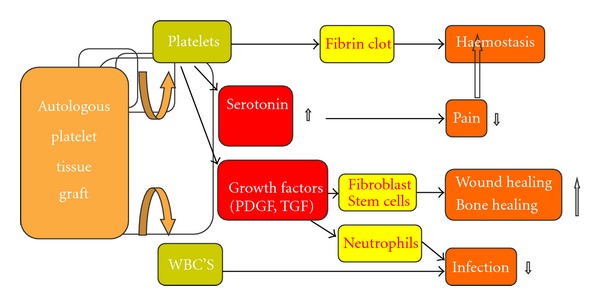
Autologous platelet tissue graft: mechanism of action.

Recently, several studies have led to the identification of growth factors that have the potential to influence the regeneration of injured muscles. Since then, multiple research groups have been trying to find drugs that work on this natural basis and can help an injured muscle to recover better and/or faster [12, 23, 24] (Figure 1). To achieve this goal, the researchers investigated several biological growth factors, such as exogenous growth factors which would promote healing of injured muscle fibers, and TGF-*β*1, the inhibition of which would block the muscle fibrosis (Table 1).

Several growth factors are capable of promoting muscle regeneration [13, 25]. These include basic fibroblast growth factor (bFGF), insulin growth factor (IGF), nerve growth factor (NGF), TGF-*β*1, and platelet-derived growth factor (PDGF). Mitchell et al. reported that the short biologic half-life of administered bFGF may limit its stimulatory effect on satellite cells [26]. They coinjected bFGF with heparin and used sustained release polymers without success. Conversely, Armand et al. found that the direct delivery of recombinant bFGF-6 into the site of injury can accelerate the regeneration of the soleus muscle in adult mice by stimulating the differentiation process of the myotubes [27].

Takahashi et al. observed that gene delivery of IGF-1 via electroporation resulted in an increased number of regenerating myofibers by 2 weeks after injury and in an increased regenerating myofiber size by 4 weeks after injury [28]. Huard et al. injected IGF in healthy old men, thus preventing the loss of muscle mass that is typical of aging. However, IGF-injection has side effects in that it promotes the development of fibrosis by stimulating the production of matrix components such as collagen and decreasing the expression of matrix-degrading enzymes such as collagenase [29]. In a mice model of muscle strain, Kasemkijwattana et al. evaluated the ability of bFGF, NGF and IGF-1 to promote muscle regeneration in vivo by three repeated injections of 100 ng into the injury site 1, 3, and 5 days after the injury [30]. In this study, physiologic strength testing was correlated with histologic analysis of the treated and nontreated muscles; the number and diameter of regenerating myofibers were monitored as an index of muscle regeneration. Their data indicated that bFGF and IGF-1, properly applied, can improve muscle performance after a strain injury. Throughout this study, growth factors had been injected on only 1 to 3 and 5 days after injury, resulting in an improvement of the tetanic and fast-twitch strength of the treated muscle, when compared with sham-injected strain-injured muscle. In addition, NGF was found capable of enhancing fast-twitch strength, but the titanic strength was not significantly different between the treated and nontreated muscle [30].

Miller et al. postulated that local delivery of HGF would augment satellite cell activation in regenerating muscle, and that this increased number of myogenic precursor cells would lead to an enhancement of muscle repair. Their study showed that, when HGF was injected in injured muscles, the number of myoblasts increased, but this increase did not lead to a better regeneration of the injected muscle. Instead, when HGF was injected the first 4 days after injury, muscle regeneration was inhibited. When it was administered later, the injection had no effect. Miller et al. also found that HGF had a dose-dependent effect on the number of myoblasts in regenerating muscles [31]. Two different doses of HGF, 6.25 and 50 ng, were used in this study. Treatment with 6.25 ng HGF did not significantly increase the number of myoblasts compared with control at any time tested. In contrast, muscles treated with 50 ng HGF on the day of injury and analyzed 1 day later yielded about threefold more MyoD-positive cells than did control muscles. In muscles further treated with HGF on subsequent days and analyzed either 2 or 3 days after injury, no significantly increased number of myoblasts was observed. This study demonstrated the effects of exogenous HGF administration on satellite cell activation and differentiation in regenerating mouse muscles after trauma. It showed the dual role HGF plays in regulating satellite cell activation and differentiation [31].

Kasemkijwattana and Menetrey et al. observed that b-FGF, IGF-1, and NGF are potent stimulators of the proliferation and fusion of myoblasts in vivo [1, 30, 32]. These growth factors were injected into mice with lacerations of the gastrocnemius muscle. Muscle regeneration was evaluated at 1 week by histological staining and quantitative histology. Muscle healing was assessed histologically and the contractile properties were measured 1 month after injury. In the treated group, the number of regenerating myofibers was increased 3.5 times for bGF and IGF-1 and 1.5 times for NGF. Those data suggested that specific growth factors were able to improve regeneration of injured muscle by stimulating myogenic proliferation and differentiation.

As discussed above, regeneration of an injured muscle consists of 2 elements. First, there needs to be proliferation and differentiation of myoblasts. This is promoted by growth factors (Table 1). Secondly, scar tissue has to be minimal. Many studies indicate that the overproduction of TGF-*β*1 is responsible for the tissue fibrosis both in animals and humans [33]. Therefore, researchers have also tried to develop drugs that inhibit TGF-*β*1. Chan et al. used the TFG-*β*1 antagonist suramin in their study. Suramin is an antiparasitic and antitumor drug that competitively binds to the TGF-*β*1 receptor. When suramin was injected immediately or 7 days after the injury, it had only a minor effect on muscle fibrosis. However, when a high dose of suramin was injected 14 days after injury, it prevented fibrosis more effectively than did a lower concentration or no suramin. There were more regenerating myofibers in all the suramin-treated groups than in the control groups. Just as the prevention of fibrosis, the number of regenerating myofibers was dose dependent. Side effects of suramin are adrenocortical insufficiency, malaise, neuropathy, and corneal deposits. Occasionally, neutropenia, thrombocytopenia, and renal failure may occur. However, the toxicity of suramin delivered via intramuscular injection has not yet been determined. In the study, no side effects were encountered [33]. These results are consistent with those of Nozaki et al. who injected 2.5 mg of suramin 2 weeks after contusion. They also found less fibrosis and better healing of the muscle. Once healed, the injected muscle was also stronger than the control muscles. A dose-response effect was not observed [34].

Decorin also inactivates the effect of TGF-*β*1. Fukushima et al. found that the injection of decorin at 10 and 15 days after injury significantly decreased the amount of fibrosis. Decorin had the additional advantage of enhancing the regeneration of the injured muscle. There seemed to be a dose-response effect. No side effects were observed [6].

## 8. Operative Treatment

Menetrey et al. used a muscle laceration model developed in mice to investigate whether surgery is a better technique to accelerate recovery of a muscle injury than immobilization. At 2 days after the laceration, the mice that had surgery only had a superficially located minor haematoma, while the immobilized mice had a larger and deeper haematoma. At the end, the immobilized mice had more and deeper scar tissue than the sutured mice. The functional results of surgery were also superior to those of immobilization [35].

Surgery can only be implemented in specific conditions. These include a large intramuscular haematoma, a complete strain or tear of a muscle with few or no agonist muscles or a partial strain if more than half of the muscle belly is torn and if the patient complains of persistent (>4–6 months) extension pain. After surgery, the operated limb should be placed in a cast and immobilized in a neutral position with an orthosis. 

## 9. Discussion

When a skeletal muscle is injured, satellite cells are activated by a variety of growth factors within 18 hours of injury, as a result of a response to a chemical stimulus [5, 36, 37]. At the same time, inflammatory cells migrate to the injury site from healthy areas of the muscle. Regeneration of single muscle fibers or entire muscles can only occur when satellite cells are activated. The optimal treatment for these muscle injuries remains obscure in routine clinical practice.

The RICE approach is generally used in the acute stage. The value of this treatment is not fully known, but most authors consider it as not harmful and maybe helpful to limit the bleeding in the muscles. It consists of rest, ice, compression, and elevation [3]. During the first 7 days after a muscle injury, rest should be taken, so that the scar tissue can gain strength. Afterwards, physiotherapy can be started [3].

Whether or not NSAIDs should be used in the treatment of muscle injuries is still controversial. They have long been the first choice to relieve pain after a skeletal muscle injury. NSAIDs may suppress the inflammatory response and thus reduce the pain and swelling. However, this response is an essential phase in the healing of injured skeletal muscle. Attempts to inhibit this phase will lead to an incomplete functional recovery. NSAIDs could interfere with macrophage action, limit phagocytic function, and impede production of growth-promoting factors that are responsible for regeneration after muscle injury. Experimental investigations showed that NSAIDs might also decrease the tensile strength of the injured muscle. Delayed muscle regeneration has been observed in treated animals [38]. Other studies did not come to this conclusion. Therefore, the exact role of NSAIDs should be established in animal models and in controlled clinical studies of skeletal muscle injuries. Until then, most authors advise that NSAIDs should not be given the first 48 hours after the injury. If the patient is in pain, paracetamol can be administered for analgesia.

A better understanding of the biological and pathological processes of muscle repair following skeletal muscle injury has led to the use of growth factors.

Growth factors have been shown to regulate satellite cell proliferation, differentiation, and fusion in myotubes in vivo and in vitro. Recently, growth factors have been found to promote the differentiation of myogenic cells in vivo and in vitro and eventually enhance complete functional recovery after muscle injury. Among these growth factors, NGF was the first to be identified and used to promote repair in peripheral and central nervous system injuries [39]. NGF may also be useful in muscle regeneration, especially during the reinnervation phase [40]. Injection of IGF increases the number and the size of regenerating myofibers after muscle injury [41]. Injection of b-FGF showed that this growth factor is a potent stimulator of the proliferation and fusion of myoblasts in vivo and in vitro [42].

TGF-*β*1 is a key factor, responsible for the formation of muscle fibrosis during the repair process by stimulating a variety of cells to increase the synthesis of numerous matrix proteins [43]. In response to muscle injury, TGF-*β*1 provides an upregulated immune mechanism which leads to an increased cellular adhesion to the ECM and ultimately enhances myofibroblast survival by inhibiting apoptosis. TGF-*β*1 is expressed at high levels and is associated with massive muscle fibrosis observed in patients with Duchenne muscular dystrophy. Based on this biological rationale of the role of TGF-*β*1, several novel researches have focused on the inhibition of TGF-*β*1 in muscle healing.

TGF-*β*1 antagonists behave as inhibitors of TGF-*β*1 by binding to its receptor and blocking its actions, in order to prevent collagen deposition and to block formation of muscle fibrosis. Among these antifibrotic agents, decorin and suramin have been demonstrated to block fibrosis and promote functional recovery of injured skeletal muscle. Decorin binds to TGF-*β*1 in order to counteract its action and suramin competitively binds to the TGF-*β*1 receptor that inhibits TGF-*β* expression [44]. However, the side effects of growth hormone factors must be taken into account, edema, and arthralgia or myalgia being most common in adults [45].

Surgery should be preserved for special cases as mentioned before. If symptoms fail to improve, the possibility of intramuscular haematoma and tissue damage should be reconsidered. Measurement of intramuscular pressure, soft-tissue X-rays, or ultrasound examination may be required [8].

## Figures and Tables

**Table 1:** Effect of growth factors in musculoskeletal tissues.

Growth factor	Skeletal muscle	Hyaline cartilage	Meniscus	Ligament	Bone
IGF-1	+	+	±	±	+
bFGF	+	+	+	±	+
NGF	+		−	−	
PDGF	−		−	±	
EGF	−	+	+	+	
TGF-alpha	−		+	−	
TGF-beta	±	+	±	±	+
BMP-2		+	+		+

^+^Positive effect; ^−^no or negative effect; blank: not tested; IGF-1: insulin-like growth factor-1; bFGF: basic fibroblast growth factor; NGF: nerve growth factor; PDGF: platelet-derived growth factor; EGF: epidermal growth factor; TGF: transforming growth factor; BMP-2: bone morphogenetic protein-2.
